# Investigating the resurgence of malaria in Rwanda: factors contributing to rising cases amidst near-eradication success and strategies for sustained control

**DOI:** 10.1097/MS9.0000000000003912

**Published:** 2025-09-17

**Authors:** Niyibizi Julius, Badaga Isimbi Diane

**Affiliations:** a Pharmacist, Kigali City, Rwanda; b Southern Medical University, School of International Education, China; c RBC, Kigali, Rwanda

**Keywords:** Anopheles gambiae, artemisinin resistance, ASPY, asymptomatic transmission, community health workers, DHAP, drone larviciding, malaria resurgence, non-pyrethroid IRS, pyrethroid resistance, Rwanda Malaria Strategic Plan

## Abstract

Rwanda is experiencing impressive control of malaria, with a reduction in cases from 4.8 million in 2016/17 to 620 000 cases by 2023/24; however, a 45.8% increase in cases by 2024 indicates an upsurge. This review analyses the reasons for this rise, using 22 sources (16 peer-reviewed papers and 6 reports) from 2022 to 2025. Major contributing factors include meteorological changes that are widening the habitable range of *Anopheles gambiae*, the emergence of pyrethroid and artemisinin resistance (K13 mutations), suboptimal coverage of non-pyrethroid indoor residual spraying (IRS), poverty and nonadherence-related disparities, and human mobility that is enabling asymptomatic transmission. Rwanda’s tentative success, with outbreaks at high altitudes and 87 000 cases in March 2024, exposes weaknesses. Suggested approaches for sustained control include community-delivered strategies using community health workers, drone-mediated larviciding (96.4% community acceptance), non-pyrethroid IRS, and screening for resistance. The 2025 Rwanda Malaria Strategic Plan highlights new drugs (dihydroartemisinin-piperaquine and artesunate-pyronaridine), regional cooperation, and partially subsidized insecticide-treated nets for at-risk populations. Gaps are *Plasmodium vivax* strategies, availability of funds, and evaluation of behavioral interventions. Elimination of lymphatic filariasis in Rwanda depends on multi-sectoral approaches that work to alleviate environmental, socioeconomic, and operational problems, cut down on recrudescence, and maintain sustained progress.

## Introduction

Rwanda has made significant progress in malaria control, with cases declining from 4.8 million in 2016/17 to 620 000 by 2023/24^[1]^. However, a 45.8% increase in cases from January to October 2024, compared to 2023, signals a concerning resurgence^[2]^. Here, we discuss the history of malaria control in Rwanda and the factors contributing to this resurgence since 2012, and also consider the susceptibility of these gains. This narrative review uses 22 sources (16 peer-reviewed papers and 6 reports) from 2022 to 2025, supplemented by contextual updates from Rwanda reputable news source. This review has explored evidence and highlighted the unique Rwandan context about ecological, socioeconomic, and operational challenges. The review, which is critical of the current literature, highlights assumptions and gaps and emphasizes the complexity of malaria transmission in the specific context of Rwanda. This article complies with the TITAN 2025 guidelines regarding the reporting and use of AI^[3]^.

**HIGHLIGHTS**

- 45.8% malaria case surge in Rwanda by 2024, despite a reduction of 620 000 cases in 2023/24.
- Climatic changes and pyrethroid/artemisinin resistance drive resurgence.
- Drone-mediated larviciding achieves 96.4% community acceptance for vector control.
- New drugs dihydroartemisinin-piperaquine and artesunate-pyronaridine were proposed in the 2025 Rwanda Malaria Strategic Plan.
- *Plasmodium vivax* strategies and funding gaps are identified as critical challenges.

## Search strategy and source selection

This narrative review employed a purposive search to identify literature on malaria’s resurgence in Rwanda, focusing on ecological, socioeconomic, and operational influences. We conducted searches on PubMed, Scopus, and Web of Science, seeking peer-reviewed articles and reports published between January 2022 and July 2025. Principal search phrases were “malaria resurgence Rwanda,” “Anopheles gambiae resistance,” “artemisinin resistance Rwanda,” and “malaria control strategies Rwanda.” We included only English-language documents that examined Rwanda’s malaria epidemiology, intervention effectiveness, and socioeconomic variables. Non-peer-reviewed material was mostly filtered out, though selective articles from *The New Times* were retained, offering timely summaries of evolving malaria dynamics and policy shifts. Additionally, national health reports from the Rwanda Ministry of Health and the World Health Organization (WHO) were incorporated to underscore strategic alignment. The final assembly comprised 22 sources (16 peer-reviewed, 6 news articles), chosen for their immediate bearing on the research question, thereby creating robust yet flexible evidence for a narrative format.

## Data analysis

This narrative review utilized a secondary data analysis approach to investigate the factors contributing to the resurgence of malaria in Rwanda and to inform strategies for sustained control. Data were sourced from 22 sources (16 peer-reviewed papers and 6 reports) from 2022–2025, retrieved from PubMed, Scopus, and Google Scholar, as well as Rwanda-specific health reports from the Rwanda Ministry of Health, WHO, and *The New Times*. The analysis involved a qualitative synthesis of evidence to categorize key factors driving malaria resurgence, including climatic and environmental changes, insecticide and drug resistance, operational and financial challenges, socioeconomic and behavioral factors, and human mobility. Quantitative data, such as malaria case trends (e.g., a reduction from 4.8 million cases in 2016/17 to 620 000 in 2023/24, followed by a 45.8% increase to 802 428 cases in 2024, including 87 000 in March alone) and intervention outcomes (e.g., 96.4% community acceptance of drone larviciding), were extracted and summarized to support the qualitative findings. The analysis directly addresses the research question by linking these factors to the resurgence of malaria and evaluating born from their implications for control strategies, as summarized in Table 1. This approach ensures a robust evidence base for understanding the complex dynamics of malaria transmission in Rwanda and identifying actionable interventions.

**Table 1:** Summary of secondary data analysis for malaria resurgence in Rwanda

Factor category	Key findings	Data source	Implication for control
Climatic/environmental	Local warming expanded the range of *Anopheles gambiae* to high-altitude areas, contributing to 2024 outbreaks (87 000 cases in March).	Hennessee *et al* (2024); Mbabazi (2025)	Scale-up drone larviciding in rice/mining areas.
Insecticide/drug resistance	K13 R561H mutation widespread by 2023; no hrp2/3 deletions in Rukara ensure diagnostic reliability.	Alruwaili *et al* (2025); Schreidah *et al* (2024)	Introduce DHAP/ASPY; enhance resistance monitoring.
Socioeconomic/behavioral	Poverty and non-adherence increase asymptomatic malaria in children under 5; low bed net use in Bugesera.	Uwimana *et al* (2025); Asingizwe *et al* (2024)	Community education and subsidized ITNs.
Operational challenges	Only 30% of high-risk areas received non-pyrethroid IRS in 2020 due to funding constraints.	Nsekuye *et al* (2024)	Increase funding for IRS and CHW programs.
Human mobility	Asymptomatic carriers in border areas sustain transmission, complicating elimination.	Mbabazi (2025); Li *et al* (2024)	Strengthen reactive case detection in border regions.

## Historical context of malaria control in Rwanda

### Early efforts and the Global Malaria Eradication Program

Malaria control efforts in Rwanda date back to the early 20th century, when, during the colonial era, the interventions used consisted essentially of quinine distribution and environmental management^[4]^. The WHO launched the Global Malaria Eradication Program (GMEP) in 1955, which promoted indoor residual spraying (IRS) using dichlorodiphenyltrichloroethane and chloroquine treatment. Rwanda, along with many other African countries, was part of the GMEP; however, due to its challenging terrain and resource limitations, the country did not achieve full coverage^[4]^. The failure of GMEP in the 1970s in sub-Saharan Africa, due to operational difficulties and the emergence of insecticide resistance, resulted in endemic malaria transmission in Rwanda^[4]^.

After GMEP, malaria control in Rwanda was based on chloroquine and sulfadoxine-pyrimethamine (SP) treatments, but resistance was reported by the 1980s^[5]^. Genocide in 1994 undermined the health system, leading to the increased burden of malaria, especially in children under 5 years^[6]^. Rwanda’s recovery of its health system in the early 2000s coincided with renewed global efforts, such as the Roll Back Malaria initiative, to introduce insecticide-treated nets (ITNs) and artemisinin-based combination therapies (ACTs)^[7]^.

### Modern interventions: ITNs, IRS, and ACTs

Rwanda scaled up ITNs, IRS, and ACTs, and by the mid-2000s, it had made major strides in reducing malaria burden^[7]^. ITN coverage reached 70% of households by 2010, and IRS was implemented in high-burden districts^[8]^. ACTs, mainly artemether-lumefantrine (coartem), became the first-line treatment, replacing SP because of the resistance^[9]^. Community health workers (CHWs) were instrumental in diagnosing and treating cases and reducing mortality from severe malaria^[10]^. Malaria incidence in Rwanda halved between 2000 and 2015, following trends observed in the WHO African Region^[4]^.

The Rwanda Malaria Strategic Plan (2019–2023) consolidated those efforts, including the use of drone-sprayed larviciding and reactive case detection, and new-generation ITNs were used as supplements to control methods^[7,11]^. There were down to 620 000 cases of malaria in 2023, compared to a peak of 4.8 million in 2016/17^[1]^. But the 2024 increase to 802 428 cases and 87 000 in March alone indicates a reversal of progress^[1,12]^.

## Factors contributing to malaria resurgence since 2012

Secondary data analysis (Table 1) identifies key factors driving the 2024 malaria resurgence in Rwanda, linking evidence from 22 sources to sustained control strategies.

### Climatic and environmental changes

As shown in Table 1, secondary data analysis links climatic changes to increased malaria transmission. Local warming has expanded the range of mosquitoes, notably of *Anopheles gambiae*, which spread into eastern Rwanda between 2010 and 2020^[8]^. Elevated temperatures and precipitation provide breeding grounds, particularly in higher-altitude, less endemic regions^[4]^. This condition is exacerbated by anthropogenic activities, including rice farming and mining, which create stagnant water bodies, which further boost the populations of mosquitoes^[11]^. The 2024 case increase in Bugesera and Gisagara is associated with environmental changes leading to more breeding sites^[2]^.

### Insecticide and drug resistance

Secondary data analysis (Table 1) highlights resistance as a key driver of resurgence. Resistance to insecticides, particularly pyrethroids, has compromised the effectiveness of IRS and ITNs. *Anopheles gambiae* in eastern Rwanda showed pyrethroid resistance in 2015 and elevated sporozoite rates and the case incidence of malaria^[8]^. Non-pyrethroid IRS, which was introduced in 2019, has contributed to the reduction of cases significantly, but its low coverage because it is costly hampers its impact^[13]^. Also, drug resistance is a concerning issue. By 2018, the artemisinin partial resistance-associated *Plasmodium falciparum* K13 R561H mutation evolved in Rwanda and was disseminated by 2023^[7]^. The k13-675V mutation and converged markers of multidrug resistance have added to the treatment complexity^[14]^. Persistent SP resistance in spite of changes to policy indicates the difficulty in so doing^[5]^. Rwanda’s plans to incorporate dihydroartemisinin-piperaquine (DHAP) and artesunate-pyronaridine (ASPY) in 2025 are an effort to combat this, but non-adherence and inappropriate drug usage may lead to subsequent resistance^[9]^.

### Operational and financial challenges

Table 1 summarizes how operational constraints, identified through secondary data analysis, contribute to resurgence. The resurgence has been driven by operational constraints such as low coverage of intervention and resource constraints. It was because of a lack of funds that only 30% of high-risk areas were sprayed with non-pyrethroid IRS in 2020^[7]^. CHW programs have been relatively cost-effective for HIV, TB, and malaria, but there has been limited research on the budget effect on governments^[10]^. However, drone larviciding, which has 96.4% community acceptability, is constrained in its scalability by cost and skills deficit ^[11]^. The 2020 National Department of Health mentored IRS in Ngoma District was not significant, in part because of uneven uptake that was affected by socioeconomic variables^[13]^.

### Socioeconomic and behavioral factors

Secondary data analysis (Table 1) underscores socioeconomic and behavioral barriers to malaria control. Resurgence has important socioeconomic determinants. Poverty and undernutrition, in particular stunting and underweight in children under 5 years old, increase asymptomatic malaria infection rates, and the cycle continues^[15]^. Rusizi District farmers and those who had difficulty accessing healthcare services were more likely to have severe malaria^[16]^. Inappropriate drug use and late seeking of medical help contribute to severe illness^[6,17]^. Insufficient bed nets, limited time, and exposure also obstruct prevention measures in Bugesera District, despite local awareness about malaria^[18]^. Resistance is being pushed by self-medication and non-adherence from the population not taking the prescribed ACTs^[2]^.

### Human mobility and asymptomatic carriers

As indicated in Table 1, secondary data analysis identifies mobility as a transmission factor. Human migration, particularly in border areas, maintains transmission. The continuous and prompt infections and asymptomatic Plasmodium carriers become reservoirs, hampering elimination^[4]^. Rwanda’s strategy of reactive case detection, which comprises testing household contacts upon diagnosis, attempts to overcome this but has its challenges in high-mobility areas^[12]^. The absence of hrp2/3 deletions in Rukara indicates that diagnostic tests remain effective, although rising k13 mutations require careful monitoring^[14]^.

## Fragility of malaria control gains

The malaria control successes of Rwanda are fragile, exemplified by the high-altitude outbreaks, as well as the 2024 epidemic. The 2012–2015 upsurge in eastern Rwanda was facilitated by *A. gambiae* resurgence, presented vulnerabilities to insecticide resistance, and climate change^[8]^. “Upscaling” in previously “safe” high-altitude areas brought disease; vector ranges expanded with rising temperatures^[4]^. The 2024 peak of 87 000 cases in March reflects several sources of pressure: mosquito adaptation to indoor measures, an uptick in outdoor spread, and resistance of parasites^[12]^. There is a case of childhood fatalities from severe malaria due to delayed treatment and socioeconomic factors^[6]^ in Eastern Province children <5 years (2017–2021).

The literature identifies shortcomings in treating *P. vivax*, which, despite being less common, complicates elimination due to hypnozoite relapse tendencies^[19]^. Current methodologies are steered toward *P. falciparum*, further emphasizing the risk of missing *P. vivax* reservoirs^[19]^. Furthermore, the plummeting effectiveness of the RTS, S vaccine and its paucity have impeded the WHO 2030 landscape targets^[20]^. Literature-based assumptions, for example, assuming uniformity in the effectiveness of interventions, do not consider Rwanda’s rich ecological and socioeconomic diversity and the need to tailor strategies.

## Strategies for sustained malaria control

### Community-based interventions

Rwanda’s strategy for malaria control involves the use of the community in its management, as highlighted by the 2025 World Malaria Day plans^[17]^. CHW programs have demonstrated cost-effectiveness for the delivery of malaria, HIV, and TB services, frequently providing better service compared to facility-based care in Low-and Middle-Income Countries (LMICs)^[1,10]^. In Rwanda, CHWs can make a diagnosis and treat cases, saving 14 380 severe deaths from malaria using the 2019 IRS campaign in Ngoma District^[13]^. The Rwanda Biomedical Center (RBC) strategy of reactive case detection that tests for household members after identifying a case aims at finding asymptomatic carriers that fuel community spread^[12]^. Awareness of the prevention of breeding sites as well as the use of ITNs through community education is on the rise, and the acceptance of drone-based larviciding has reached 96.4% in Kigali^[11]^.

However, socio-economic factors such as poverty and low access to healthcare services decrease the effectiveness of the CHW program in remote areas such as Rusizi District, where severe malaria prevalence remains high, reaching 64.03%^[16]^. The studies recommend that CHW services be linked with income-generating interventions such as farming cooperatives to contribute to addressing poverty-related barriers^[18]^. There are still some gaps in the evidence on the affordability of scaling CHW programs and limited evidence on budget impacts on the health system and government^[10]^. Suggested strategies in the future should prioritize communities’ mobilization on education-based campaigns and incentives as a way to improve intervention adherence. The secondary data analysis (Table 1) supports CHW programs and reactive case detection as effective, despite socioeconomic barriers^[10,12,16]^.

### Innovative technologies and vector control

Rwanda adopted new technologies to fight against the resurgence of malaria. Drones applied larviciding with *Bacillus thuringiensis var. israelensis* (Bti) for Kigali City, demonstrating high community acceptance and effectiveness of controlling mosquito breeding^[11]^. This focuses on rice growing areas and mining areas, with the particular risk of large areas of stagnant water in which *A. gambiae* can flourish^[8]^. Non-pyrethroid IRS intervention implemented in 2019 considerably contributed toward reducing malaria incidence in Eastern Rwanda through mitigation of pyrethroid resistance^[13]^. Furthermore, new generation ITNs with two insecticides have been implemented since 2018, aimed at preventing insecticide resistance^[7]^. The impacts of these innovative vector control interventions, implemented between 2018 and 2021, are summarized in Table 2.

**Table 2:** Key interventions and their impact (2018–2024)

Intervention	Impact
Non-pyrethroid IRS (2019)	Reduced malaria incidence in Eastern Rwanda by addressing pyrethroid resistance.
Drone-based larviciding (2019)	Controlled mosquito breeding in high-risk areas with 96.4% community acceptance.
Dual-insecticide ITNs (2018)	Improved protection against resistant mosquitoes in high-burden districts.

Beyond ITNs and IRS, novel vector control approaches hold potential. Enhanced housing design with screened (mosquito-proof) windows, as well as sealed eaves, limits mosquito penetration and supplements control measures that are already in place^[21]^. Spatial repellents, such as plant-derived oils, address outdoor transmission, which has escalated as a result of mosquito behavioral resistance^[2,21]^. Yet, in scaling up these technologies, there are barriers to overcome, such as their relatively high costs and the limited local capacity of drone operators^[11]^. The literature emphasizes the importance of novel financing: public–private partnership and sustainability^[11]^. Rwanda provides an example that as these technologies get mainstreamed in national approaches, community education becomes even more important for sustaining trust and participation.

### Enhanced surveillance and molecular monitoring

Strong surveillance is also necessary to keep malaria in check as resistance to drugs and insecticides rises. Rwanda started its 2025 surveillance efforts on World Malaria Day, centered on early detection and molecular resistance marker monitoring^[17]^. K13 R561H and k13-675V mutations of *P. falciparum* associated to artemisinin partial resistance became widespread from 2018, with a need for continuous monitoring^[7,14]^. The fact that there are no hrp2/3 deletions in Rukara offers reliability of diagnostic testing and monitoring the course of treatment, but continuing resistance to SP emphasizes updating of treatment recommendations^[5,14]^.

In Rwanda, a Multiple First-Line Treatment (MFT) strategy was tested in 2024, wherein three ACTs were used in rotation for the purpose of delaying resistance^[22]^. New impetus and investment are anticipated in the treatment of resistant parasites in 2025 when two new drugs, DHAP and ASPY, are launched, with the concurrent release of updated treatment regimens and healthcare worker training^[9]^. Yet, non-adherence and self-medication threaten to overshadow these enterprises, as evidenced by the case surge for 2024^[2]^. The literature suggests that it is also possible to combine surveillance with active case detection to detect the asymptomatic carriers, with particular focus on border regions with high levels of mobility^[12]^. Deficits in surveillance of *P. vivax*, which could stand as cryptic reservoirs, call for surveillance across the species^[19]^. The analysis of resistance data in Table 1 emphasizes the need for K13 mutation monitoring and new drugs (DHAP, ASPY)^[5,9]^.

### Policy adaptations and regional collaboration

New approaches taken by Rwanda on this issue cut across complex resurgence challenges. The Rwanda Malaria Strategic Plan (2019–2023) led to a 76% reduction in incidence from 2018 to 2022, but rising resistance and resource limitations require updates^[7,22]^. The 2025 strategy focuses on community engagement, with new drugs and surveillance, and is targeting a 75% reduction in prevalence by 2025^[1]^. Selected vulnerable groups, including under-five children and farmers, benefit from subsidized ITNs and early treatment for severe malaria to reduce mortality^[6,16]^. This has added to the list of undernutrition, which is a co-factor, and other domestic policies are required to be put in place, which all enforce to elevate food security and including health and agriculture policies in the process^[15]^.

There is much to be learned from regional and global cooperation. China’s malaria elimination by 2020 via integrated vector management and mass drug administration is a model for Rwanda^[4]^. If China and Africa cooperate better in such areas, this will increase Rwanda’s access to new drugs and vaccines (like R21/Matrix-M, which, although promising, is currently facing distribution challenges)^[4,20]^. Nevertheless, the literature also “criticizes” dependence on vaccines such as RTS, S that have tenuous efficacy and availability^[20]^. Collaboration by Rwanda in regional efforts like the East Africa Community’s response to malaria control efforts could strengthen cross-border surveillance and resource sharing that confront transmission due to human mobility^[4]^.

## Addressing socioeconomic and behavioral barriers

Malaria control success is also profoundly affected by socioeconomic and behavioral factors. Poverty, no bed net, and late health seeking lead to a higher risk of severe malaria, in particular in Eastern Province^[6,18]^. Rwanda’s community-based education in proper medication use and environmental management has increased preventive behaviors in Bugesera District^[18]^. Support from the family and mobilization of the community are important drivers for progress, although lack of time and misinformation act as barriers^[2,18]^.

They demonstrated a significant positive correlation between the average productivity on a farm and both the ownership of mosquito nets and their appropriate use for malaria control.

### Economic analyses

Several economic analyses based on Kenya’s Malaria Strategy suggest that the control of malaria affects the labor supply and GDP in a country, thereby justifying an increase in government expenditure^[23]^. Rwanda could follow both of these models, making investments in health infrastructure and CHW training to lower costs in the long term. Interventions directed at social determinants, for example, improving housing and service access in rural settings, are consistent with what is described as integrated vector management^[21]^. The literature reveals a lacuna in the economic evaluation of behavioral interventions and highlights the requirement for mixed-methods research to inform community engagement^[18]^.

## Critical evaluation and gaps

Literature offers strong evidence in favor of the approaches adopted by Rwanda, with some inconsistencies. Assumptions based on uniform improvement of interventions appear to underestimate the ecological diversity of Rwanda, which includes regulations of diffusion reviewers^[4]^. The emphasis on *P. falciparum* excludes *P. vivax*, requiring separate elimination strategies because of hypnozoite relapse^[19]^. Spending on the scalability of drone-based larviciding and CHW programs is scant, presenting strategic challenges^[10,11]^. The declining effectiveness of RTS, S and its delayed deployment highlight the urgency for a locally made vaccine, a need for the sub-Saharan African space^[20]^.

Over-dependence on external funding to support interventions like IRS and ITNs may lead to sustainability challenges, as evidenced by the lower effectiveness of the 2020 Ngoma campaign^[13]^. The published studies are not extended into the long-term use of new drugs such as DHAP and ASPY due to fast resistance^[9]^. Behavioral interventions hold potential but need to be evaluated^[18]^ for combating misinformation and non-adherence. Cost–benefit analyses, surveillance of *P. vivax*, and association with socio-economic interventions are recommended to guarantee access to control tools.

## Future directions

Future research should prioritize: (1) mixed-methods studies to evaluate the cost-effectiveness and scalability of drone larviciding and CHW programs, addressing funding constraints^[11]^; (2) epidemiological studies on *P. vivax* prevalence and relapse prevention strategies to complement *P. falciparum*-focused efforts^[19]^; (3) economic analyses of behavioral interventions to improve adherence and reduce misinformation in high-risk districts like Bugesera^[17]^; and (4) development and evaluation of locally produced vaccines to enhance availability and sustainability, reducing reliance on external funding^[20]^.

## Conclusion

The trajectory of Rwanda to malaria elimination by 2030 depends on the added array of interventions to tackle the 2024 resurgence. Community-based interventions using CHWs and reactive case detection improve early case detection and management and innovative tools, such as drone-based larviciding and non-pyrethroid IRS target vector resistance. Better surveillance and better medicines fight drug resistance, but that drug resistance, as well as socioeconomic obstacles and *P. vivax* gaps, need to be addressed. Regional cooperation, emulating the success of the China model, and policy adjustments focusing on the vulnerable groups add weight to Rwanda’s strategy. But sustainability rests on addressing funding limitations, examining the affordability of the intervention, and prioritizing locally made vaccines. By combining these approaches, the secondary data analysis (Table 1) links the 2024 resurgence to multiple factors, supporting strategies like non-pyrethroid IRS and drone larviciding. Rwanda can turn the resurgence back and maintain control gains, maintaining momentum after a period of unprecedented achievement.

## Data Availability

All relevant data used in this study are provided within the manuscript. Data sharing is not applicable to this article, as no new datasets were generated or analyzed. The study is a narrative review based on publicly available peer-reviewed literature.

## Research registration unique identifying number (UIN)

This study is a narrative review and does not involve human subjects or primary research data collection, so registration is not required.

## References

[1] Mbabazi J. *Rwanda to focus on community action in malaria fight*, in *The New Times*. Thursday, April 24, 2025.

[2] Ntirenganya E. *Minister urges redoubled prevention efforts as Malaria cases surge*, in *The New Times*. Saturday, January 04, 2025.

[3] Agha RA Mathew G Rashid R. Transparency in the Reporting of Artificial Intelligence – the TITAN guideline. Prem J Sci 2025;2. https://www.researchgate.net/publication/392003420_Transparency_In_The_reporting_of_Artificial_INtelligence_-_the_TITAN_guideline

[4] Li J Docile HJ Fisher D. Current status of malaria control and elimination in Africa: epidemiology, diagnosis, treatment, progress and challenges. J Epidemiol Glob Health 2024;14:561–79. 38656731 10.1007/s44197-024-00228-2 PMC11442732

[5] Alruwaili M Elderdery A Manni E. A narrative review on the prevalence of *plasmodium falciparum* resistance mutations to antimalarial drugs in Rwanda. Trop Med Infect Dis 2025;10:89. 10.3390/tropicalmed10040089 40278762 PMC12030788

[6] Hategekimana JP Simbi CMC Ntakirutimana T. Factors associated with severe malaria-related mortality among hospitalized children under five years of age in Eastern Province of Rwanda: a cross-sectional study using hospital records from 2017 to 2021. Malar J 2024;23:340. 39529067 10.1186/s12936-024-05159-8 PMC11555802

[7] Umugwaneza A Mutsaers M Ngabonziza JCS. Half-decade of scaling up malaria control: malaria trends and impact of interventions from 2018 to 2023 in Rwanda. Malar J 2025;24:40. 39934796 10.1186/s12936-025-05278-w PMC11817622

[8] Ian Hennessee P Alphonse M Munyakanage D. BS2, Dunia Munyakanage, MPH2, Michee Kabera, MPH2, M. Aimable Mbituyumuremyi, Naomi Lucchi, PhD3,4, Miles A. Kirby, PhD5, Lance A. Waller, PhD1,6, and P. Thomas F. Clasen, Uriel Kitron, PhD1,7, & Emmanuel Hakizimana, PhD2. Am J Trop Med Hyg 2024;112:56–65. 39471513 10.4269/ajtmh.23-0881 PMC11720781

[9] Ntirenganya E. *Rwanda to introduce new drugs to combat malaria resistance*, in *The New Times*. Monday, January 06, 2025.

[10] O’Donovan J Baskin C Stansert Katzen L. Costs and cost-effectiveness of community health worker programs focussed on HIV, TB and malaria infectious diseases in low- and middle-income countries (2015-2024): a scoping literature review. PLOS Glob Public Health 2025;5:e0004596. 40343952 10.1371/journal.pgph.0004596 PMC12063845

[11] Munyakanage D Niyituma E Mutabazi A. Factors affecting community participation in drone-based larviciding using Bacillus thuringiensis var. israelensis (Bti) for bio-control of malaria vectors in Rwanda. Malar J 2025;24:67. 40025519 10.1186/s12936-025-05310-z PMC11872309

[12] Mbabazi J. *Rwanda rolls out new malaria strategy after recording 87,000cases in March*, in *The New Times*. Wednesday, April 23, 2025.

[13] Nsekuye O Malamba SS Omolo J. Indoor residual spraying uptake and its effect on malaria morbidity in Ngoma district, Eastern province of Rwanda, 2018-2021. Malar J 2024;23:381. 39696480 10.1186/s12936-024-05194-5 PMC11658272

[14] Schreidah C Giesbrecht D Gashema P. Expansion of artemisinin partial resistance mutations and lack of histidine rich protein-2 and −3 deletions in *Plasmodium falciparum* infections from Rukara. Rwanda Malar J 2024;23:150. 38755607 10.1186/s12936-024-04981-4 PMC11100144

[15] Uwimana A Robert A Ahmed A. Exploring the prevalence and association between nutritional status and asymptomatic malaria in Rwanda among under-5 children: a cross-sectional analysis. Malar J 2025;24:152. 40361133 10.1186/s12936-025-05370-1 PMC12077043

[16] Uwamahoro B Munyanshongore C Ndagijimana A. Factors associated with severe malaria among patients under reference to district hospitals: a cross-sectional study in Rusizi District. Rwanda. Rwanda J Med Health Sci 2023;6:36–42. 40567925 10.4314/rjmhs.v6i1.5 PMC12110463

[17] Mbabazi J. *Rwanda launches new malaria surveillance, treatment programmes*, in *The New Times*. Saturday, April 26, 2025.

[18] Asingizwe D Tuyizere M Mukeshimana M. Why becoming a positive deviant for malaria prevention and control: a sequential explanatory mixed methods study in Bugesera district. Rwanda Malar J 2024;23:284. 39300522 10.1186/s12936-024-05108-5 PMC11414207

[19] Quaye IK Aleksenko L Paganotti GM. Malaria elimination in Africa: rethinking strategies for *Plasmodium vivax* and lessons from Botswana. Trop Med Infect Dis 2023;8:392. 37624330 10.3390/tropicalmed8080392 PMC10458071

[20] Oladipo HJ Tajudeen YA Oladunjoye IO. Increasing challenges of malaria control in sub-Saharan Africa: priorities for public health research and policymakers. Ann Med Surg (Lond) 2022;81:104366. 36046715 10.1016/j.amsu.2022.104366 PMC9421173

[21] Nalinya S Musoke D Deane K. Malaria prevention interventions beyond long-lasting insecticidal nets and indoor residual spraying in low- and middle-income countries: a scoping review. Malar J 2022;21:31. 35109848 10.1186/s12936-022-04052-6 PMC8812253

[22] Kuteesa H. *Malaria drug resistance: Rwanda updates treatment guidelines*, in *The New Times*. Wednesday, July 24, 2024.

[23] Elnour Z Grethe H Siddig K. Malaria control and elimination in Kenya: economy-wide benefits and regional disparities. Malar J 2023;22:117. 37029370 10.1186/s12936-023-04505-6 PMC10080938

